# C-755-06. Burns Amplify Risk of Stroke and Thromboembolic Events in Hypertension and Type 2 Diabetes Patients

**DOI:** 10.1093/jbcr/irag033.042

**Published:** 2026-04-06

**Authors:** Snehal A Kumar, Elsa Chittet, Amina El Ayadi, Steven E Wolf, Juquan Song

**Affiliations:** University of Texas Medical Branch, John Sealy School of Medicine, Galveston, TX; UNT Health Fort Worth - Texas College of Osteopathic Medicine, Sugar Land, TX; Division of Burn and Trauma Surgery, Department of Surgery, University of Texas Medical Branch, Galveston, TX; Division of Burn and Trauma Surgery, Department of Surgery, University of Texas Medical Branch, Galveston, TX; University of Texas Medical Branch, Galveston, TX

## Abstract

**Introduction:**

Hypertension (HTN) and type 2 diabetes (T2DM) are common chronic diseases that significantly increase the risk of stroke. Burn-induced coagulopathy and inflammation create a prothrombotic state, with elevated stroke risk persisting well beyond initial recovery. This study investigates the impact of burn trauma on the incidence of stroke and other thromboembolic events in patients with chronic conditions.

**Methods:**

We conducted a retrospective cohort study using TriNetX, a real-world health data platform. Adult HTN and T2DM patients diagnosed pre-2014 across 70 U.S. healthcare organizations were stratified by the presence or absence of a burn injury within one year of the comorbid diagnosis. Cohorts were 1:1 propensity-score matched (PSM) on 37 covariates, including demographics, malignancies, and other confounders. Vascular outcomes over 1, 5, and 10 years were assessed using risk ratios (RR), hazard ratios (HR), and Kaplan–Meier analysis. 95% confidence intervals (CI) and significant *p*-values (< 0.05) were reported. Methods were repeated in a second, 2015-2021 study of patients aged 65-90 at the index diagnosis to evaluate 1- and 2-year outcomes in elderly populations.

**Results:**

After PSM, cohort sizes varied from 5244 to 12 604 patients per group. Burn injury was associated with higher ischemic stroke risk in both pre-2014 HTN (RR 1.30, CI 1.05-1.62) and T2DM (RR 1.61, CI 1.21-2.15) groups at 5 years. Similar elevations were noted at 10 years (HTN: RR 1.29, CI 1.11-1.50; T2DM: RR 1.30, CI 1.07-1.58). Transient ischemic attack (TIA) risk was also increased in HTN patients 10 years post-burn (RR 1.40, CI 1.13-1.74) and in T2DM patients 5 years post-burn (RR: 1.628, CI 1.07-2.48). In the elderly cohorts, TIA risk was elevated in HTN patients 2 years post-burn (RR 1.69, CI 1.07-2.67). Kaplan–Meier analyses supported these associations. Other thromboembolic outcomes, including deep vein thrombosis (DVT) and pulmonary embolism (PE), were significant in certain HTN and T2DM time-interval comparisons.

**Conclusions:**

Burn injury combined with a HTN or T2DM diagnosis was associated with statistically significant increases in ischemic stroke and TIA 5 and 10 years post-injury. Findings were consistent across adult and elderly populations, underscoring the long term risk of cerebrovascular accidents in burn survivors with chronic conditions.

**Applicability of Research to Practice:**

Burn survivors with comorbid diagnoses require sustained monitoring and preventative care to mitigate elevated cerebrovascular risks.

**Funding for the study:**

N/A.

